# RETRACTION: Acute Toxicity Study of Zerumbone‐Loaded Nanostructured Lipid Carrier on BALB/c Mice Model

**DOI:** 10.1155/bmri/9819461

**Published:** 2025-09-09

**Authors:** BioMed Research International

RETRACTION: H. S. Rahman, A. Rasedee, H. H. Othman, et al., “Acute Toxicity Study of Zerumbone‐Loaded Nanostructured Lipid Carrier on BALB/c Mice Model,” *BioMed Research International* 2014, (2014): 563930, https://doi.org/10.1155/2014/563930.

The above article, published online on 08 September 2014 in Wiley Online Library (http://wileyonlinelibrary.com), has been retracted by agreement between the Journal Section Editor, Jinsong Ren, and John Wiley & Sons Ltd. The retraction has been agreed due to multiple concerns with the figures presented in the manuscript, initially raised on PubPeer [1]. On further investigation, additional issues were found:

• Figure 2a contains unexpected overlap between panels D and E
• Figure 3a panel H contains unexpected overlap with Figure 3b panel A
• Figure 4a contains unexpected overlap between panels B and F
• Figure 4a panel H contains unexpected overlap to Figure 4b panel G
• Figure 4b contains unexpected overlap between panels A and C
• Figure 5a contains unexpected overlap between panels B and C
• Figure 5b contains unexpected overlap between panels B and G
• Figure 6a contains unexpected overlap between panels A and E
• Figure 6a contains unexpected overlap between panels B and C
• Figure 6b contains unexpected overlap between panels B and F
• Figure 6b contains unexpected overlap between panels B and C
• Figure 6b contains unexpected overlap between panels C and F
• Figure 6b contains unexpected overlap between panels D and E
• Figure 7a panel G contains unexpected overlap with Figure 7b panel A
• Figure 7b contains unexpected overlap between panels B and C
• Figure 7b contains unexpected overlap between panels D and E

The authors did not provide an adequate response or supporting data to address the concerns, and therefore the article is retracted with agreement by the editorial board.

Heshu Sulaiman Rahman, Abdullah Rasedee, Hemn Hassan Othman, Max Stanley Chartrand, Nozlena Abdul Samad and Chee Wun How disagree with the retraction. The remaining authors were unresponsive.
